# Cerebral clues: serum neurofilament light chain (sNfL) as a novel biomarker for immune check point inhibitor (ICI) mediated seronegative encephalitis

**DOI:** 10.1093/omcr/omae058

**Published:** 2024-06-07

**Authors:** Anza Zahid, Sudhakar Tummala

**Affiliations:** Department of Neurology, Houston Methodist Hospital, Houston, TX 77030, United States; Department of Neuro-Oncology, The University of Texas MD Anderson Cancer Center, Houston, TX 77030, United States

**Keywords:** Immune checkpoint inhibitors, encephalitis, neurofilament light chain

## Abstract

Immune checkpoint inhibitor (ICI) mediated encephalitides are increasingly being recognized in the literature, but atypical cases may be missed or misdiagnosed. Recent efforts are directed to identify biomarkers to help elucidate early diagnosis and treatment. Herein, we describe two cases of antibody negative ICI-mediated encephalitis with elevated serum Neurofilament light chain (sNfL) levels. Practical Implication: Baseline and longitudinal measurements of serum neurofilament light chains can help determine treatment strategies and prognosis in patients with suspected immune checkpoint inhibitor encephalitis.

## Introduction

Immune checkpoint inhibitor (ICI) mediated encephalitides are associated with increased morbidity and mortality [1]. These toxicities are being increasingly recognized in the literature, but atypical cases may be missed or misdiagnosed.

Recent efforts are directed to identify biomarkers to help elucidate early diagnosis and treatment. Neurofilament light chain (NfL) is a neuronal cytoplasmic protein that is highly expressed in dendrites, neuronal soma, large caliber myelinated axons in the cerebrospinal fluid (CSF) and blood, proportionally [2]. Under normal condition NfL is highly stable in the axons with low turnover into the CSF and blood in an age dependent manner [3]. Over the last two decades, NfL has been studied as a novel biomarker of neuroaxonal injury in demyelinating disease, neurodegenerative diseases, and autoimmune encephalitis [2, 3].

Herein, we described two cases of antibody negative ICI-mediated encephalitis with serum neurofilament light chain testing.

## Methods

### Standard protocol approvals, registrations, and patient consents

Institutional Review Board under IRB ID 2022-1055 granted exemption for study.

### Case reports

We retrospectively identified patients that were seen by the Neuro-Oncology inpatient-consult service at MD Anderson from January 2015—August 2023. Two patients met the criteria of 1.) neurological onset of symptoms after initiation of immune checkpoint inhibitors, 2.) on whom serum neurofilament light chain levels (sNfL) were obtained for suspected immune mediated encephalitis and 3.) serology for encephalitis antibodies. A modified Rankin score (mRS) is utilized to measure neurological disability in both cases.

### Case 1

A 71-year-old male with epithelioid neoplasm of the lung presented with confusion, personality changes, and loss of appetite within 2 weeks after cycle 6 of pembrolizumab. Upon admission, laboratory findings demonstrated acute kidney injury, hypernatremia (149), and mild hypokalemia (3.1) and low cortisol and ACTH levels. The initial examination revealed inattention and lethargy. He was able to follow simple commands and move all extremities with intact motor strength and reflexes bilaterally. He was diagnosed with ICI-mediated central adrenal insufficiency (CAI) and treated with steroids and thiamine. For his intermittent inattention the diagnosis of toxic metabolic encephalopathy was favored. However, the patient became more delirious and lethargic despite correction of metabolic derangements. On repeat exam, he was only able to follow 1-step commands intermittently, recognize few objects, unable to calculate change, and able to move extremities spontaneously. Routine electroencephalogram (EEG) demonstrated mild diffuse slowing without epileptiform discharges. Brain MRI with and without contrast demonstrated no signal abnormalities. Lumbar puncture revealed protein of 44, glucose of 86, white blood cells (WBC) of 1 and red blood cells (RBC) of 1. The infectious panel and cytology were negative. Cerebral spinal fluid (CSF) studies and serum autoantibody panels were negative. Serum neurofilament light chain was found to be elevated (326 pg/ml (normal <37.9 pg/ml). The patient was started empirically on plasmapheresis for clinical suspicion of sero-negative ICI-mediated encephalitis. The patients showed gradual cognitive improvement with plasmapheresis and steroids. He was seen in clinic one month after discharge with significant improvement. He demonstrated only mild residual slowing while performing complex-task. Repeat serum neurofilament light chain level was normal (33.3 pg/ml). Five months since the onset of his symptoms, his mRS is 2.

### Case 2

**Figure 1:**
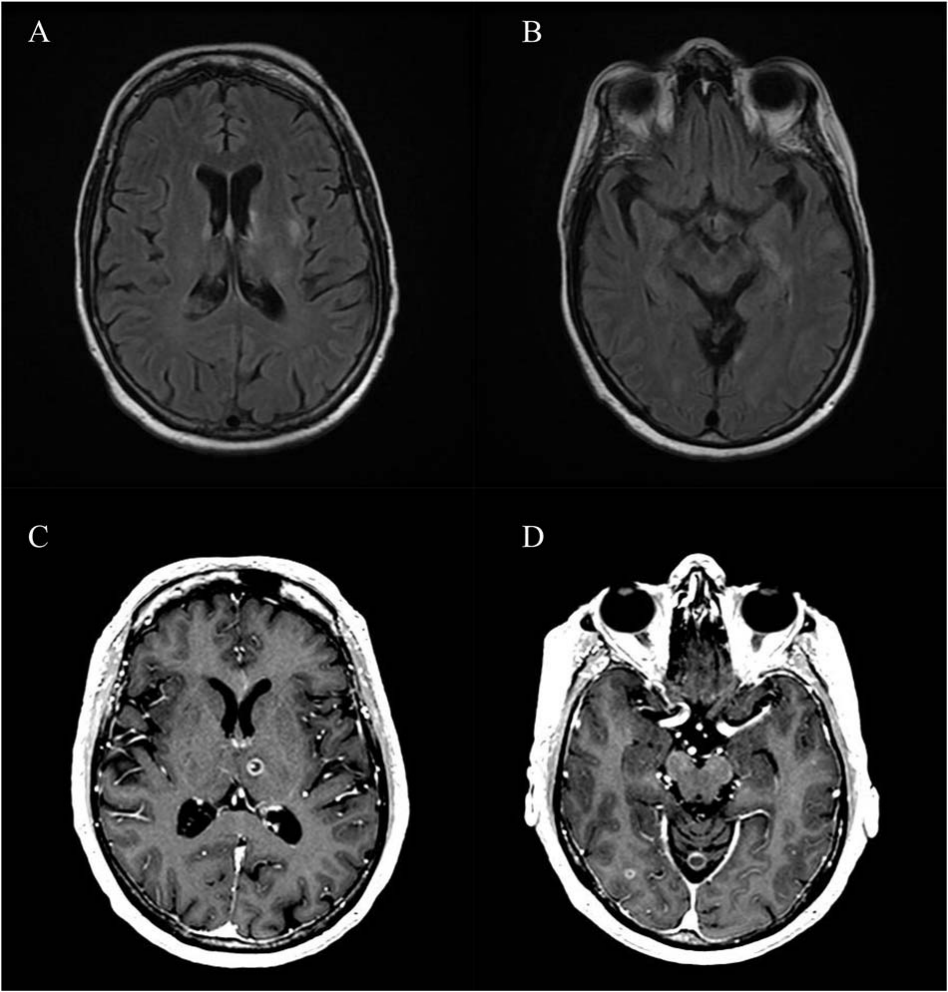
Scattered FLAIR signal hyperintensities (A and B) and T1 post-contrast enhancing metastatic lesions post-gamma knife radiations (C and D).

**Figure 2:**
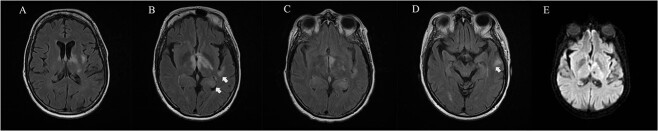
Progression of scattered FLAIR signal hyperintensities in the hypothalamic region extending along the left thalamic capsule (A and B), parahippocampal region on the left, right temporal and left temporal lobes (C and D), and along the occipital horns (D). Subtle diffusion restriction along the thalamic and hypothalamic region (E).

A 62-year-old female with stage-IV adenocarcinoma of the lung and brain metastasis presented to the hospital with worsening alerted mentation after completing 1 cycle of nivolumab and ipilimumab. On the first day of Cycle 2, she reported marked fatigue and diarrhea hence, the immunotherapy was held. Five days later, the patient was brought in by family for altered mental status. On the exam, she was awake alert, and oriented to time, place, and person. Intact motor and sensory testing. The mRS at admission was 3. Brain MRI demonstrated stable known brain metastasis post-gamma knife irradiation (Fig. 1). The patient continued to decline with worsening cognition, prolonged episodes of aphasia and a new progressive right-sided weakness. Repeat Brain MRI showed new increased T2 FLAIR signal intensity in hypothalamic and parahippocampal regions and subtle enhancements in the occipital regions (Fig. 2B–D). Some of the above lesions showed subtle areas of restricted diffusion in subcortical regions (Fig. 2E). Routine EEG showed diffuse slowing without epileptiform discharges. Lumbar puncture revealed lymphocytic pleocytosis with protein 60 mg/dl, 68 WBC, 3 RBC without evidence of metastatic disease favoring ICI-mediated limbic encephalitis. Cerebrospinal fluid studies were negative for paraneoplastic autoimmune antibody panel. Serum NfL level was elevated to 111 pg/ml (normal <28 pg/ml). The patient was started on steroids and plasmapheresis with only transient improvement in verbal response. The patient was readmitted 30 days later with infections (pneumonia, urinary tract infections) and worsening mentation. Repeat EEG showed toxic metabolic features without evidence of seizures. Lumbar puncture was repeated twice that showed persistent lymphocytic pleocytosis without evidence of malignancy, with normalization of CSF protein. However, the patient continued to decline with poor neurological exam. Repeat sNfL levels showed an increase (182 pg/ml). T cell suppressive therapy with tacrolimus was initiated [4, 5]. Clinically, the patient remained semi-comatose with being able to withdraw on the left upper extremity only. Further work up and treatment was not pursued per family’s request during the readmission. The patient was transitioned to hospice care and expired due to sepsis.

## Discussion

Serum NfL levels are highly sensitive markers to measure neuroaxonal damage in various neurological diseases such as neurodegenerative, strokes, and multiple sclerosis [2, 6].

Immune checkpoint inhibitors can cause concomitant organ toxicities such as immune mediated adrenal insufficiency and thyroiditis that may present with altered mentation. We show that serum neurofilament light chain (sNfL) could serve as a novel biomarker for immune checkpoint inhibitor (ICI) mediated autoimmune encephalitis when an alternative diagnosis is in question. In this study, we identified atypical cases of autoimmune encephalitis where a diagnosis of toxic metabolic encephalopathy (case 1) and subacute strokes (case 2) was favored. Serum NfL levels were found to be sharply elevated (normal range: <4.62 pg/ml and <7.65 pg/ml adjusted for age 60 to 69 years and 70 to 79 years, respectively) [7].

In case 1, the initial presentation was consistent with toxic metabolic encephalopathy with underlying ICI-mediated central adrenal insufficiency (CAI). Despite correction of metabolic derangements, there was no clinical improvement. Elevated sNfL suggested neuroaxonal injury. Steroids and plasmapheresis were initiated early. The longitudinal normalization of sNfL level mirrored an improvement in the mRs observed in case 1. This supports early identification and treatment initiation may result in early clinical recovery in autoimmune encephalitis [8].

In case 2, the patients’ cognitive decline promoted initial evaluation for subclinical seizures, strokes, and toxic metabolic encephalopathy. Although MRI Brain showed some areas with restricted diffusion in the temporal lobe and hippocampal region (Fig. 1C), the pattern of diffusion restriction was atypical for subcortical vascular territorial strokes. Ictal phenomenon can cause cortical diffusion restriction however, there were no ictal foci or events captured on the EEG. Budhram et al. compared patterns of diffusion restriction in patents with peri-ictal medial temporal lobe and limbic encephalitis. Diffuse hippocampal diffusion restriction sparing medial temporal lobe structure (Pattern 2) were observed in patients with limbic encephalitis (P = 0.0001) [9]. Additionally, no significant differences have been observed in NfL concentrations in patients with versus without brain metastasis [10]. The lumbar puncture that revealed persistent pleocytosis suggested an inflammatory process. Median sNfL range in acute ischemic strokes is 16.7–21.0 pg/ml in patients with National Institutes of Health Stroke Scale (NIHSS) of 7−>15 (P = 0.01) that did not correspond to levels observed in case 2 [11]. Prolonged status epilepticus and drug resistant epilepsy may be associated with neuroaxonal damage leading to elevation of neurofilament levels [12]. However, repetitive injury secondary to refractory status and an EEG correlation is necessary to attribute elevation of levels to seizures [12].

Immune checkpoint inhibitors mediate CD 8+ T-cell dominant CNS inflammatory response with perivascular lymphocytic infiltration, reactive astrogliosis, and neuroaxonal loss. It may result in blood brain barrier leakage leading to extravasation of neurofilament light chain [13]. Inflammatory process mediated by CD 8+ T cells may result in irreversible neuronal injury; hence; early identification and management is warranted [13, 14]. Therefore, serum NfL may be utilized as a marker of central nervous system damage and parameter for treatment in suspected immune-mediated encephalitis, when patients do not present with a defined syndrome and in absence of paraclinical findings, such as stable or negative MRI findings, lack of seizures, or autoantibody detection assays as observed in our patients [10].

Although, serum NfL levels may decrease with treatment with plasmapheresis and concomitant immunoglobulins however, the paucity of large prospective and retrospective data makes it challenging to conclude whether there is significant reduction by removal alone [15]. In multiple sclerosis, a decrease in NfL may indicate reduced neural inflammation, restoration of blood brain barrier integrity, and treatment efficacy [16].

Two measurements of serum NfL could have prognostic utility: a baseline measurement—levels obtained at the disease onset, and longitudinal measurements for monitoring clinical outcomes and treatment response. Further large prospective studies are warranted to determine independent determinants of neuroaxonal injury in ICI-mediated encephalitis and relatedness of elevated NfL levels in these patients.

## CONFLICT OF INTEREST

The authors declare that they have no conflicts of interest.

## FUNDING

None declared.

## References

[1] Vogrig A, Muñiz-Castrillo S, Joubert B. et al. Central nervous system complications associated with immune checkpoint inhibitors. J Neurol Neurosurg Psychiatry 2020;91:772–8. 32312871 10.1136/jnnp-2020-323055

[2] Gaetani L, Blennow K, Calabresi P. et al. Neurofilament light chain as a biomarker in neurological disorders. J Neurol Neurosurg Psychiatry 2019;90:870–81. 30967444 10.1136/jnnp-2018-320106

[3] Khalil M, Teunissen CE, Otto M. et al. Neurofilaments as biomarkers in neurological disorders. Nat Rev Neurol 2018;14:577–89. 30171200 10.1038/s41582-018-0058-z

[4] Liu C, Ji S, Gao H. et al. Efficacy of tacrolimus as long-term immunotherapy for neuronal surface antibody-mediated autoimmune encephalitis. Ther Adv Chronic Dis 2022;13:204062232110630. 10.1177/20406223211063055 PMC8755929 35035868

[5] Xiao J, Fu PC, Li ZJ. Clinical and imaging analysis to evaluate the response of patients with anti-DPPX encephalitis to immunotherapy. BMC Neurol 2022;22:129. 35382765 10.1186/s12883-022-02649-7 PMC8981927

[6] Körtvelyessy P, Prüss H, Thurner L. et al. Biomarkers of neurodegeneration in autoimmune-mediated encephalitis. Front Neurol 2018;9:668. 10.3389/fneur.2018.00668. 30283395 PMC6156245

[7] Vermunt L, Otte M, Verberk IMW. et al. Age- and disease-specific reference values for neurofilament light presented in an online interactive support interface. Ann Clin Transl Neurol 2022;9:1832–7. 36196979 10.1002/acn3.51676 PMC9639622

[8] Graus F, Titulaer MJ, Balu R. et al. A clinical approach to diagnosis of autoimmune encephalitis. Lancet Neurol 2016;15:391–404. 26906964 10.1016/S1474-4422(15)00401-9 PMC5066574

[9] Budhram A, Britton JW, Liebo GB. et al. Use of diffusion-weighted imaging to distinguish seizure-related change from limbic encephalitis. J Neurol 2020;267:3337–42. 32583056 10.1007/s00415-020-10007-1

[10] Bjursten S, Zhao Z, Al Remawi H. et al. Concentrations of S100B and neurofilament light chain in blood as biomarkers for checkpoint inhibitor–induced CNS inflammation. EBioMedicine 2024;100:104955. 38171113 10.1016/j.ebiom.2023.104955 PMC10796943

[11] De Marchis GM, Katan M, Barro C. et al. Serum neurofilament light chain in patients with acute cerebrovascular events. Eur J Neurol 2018;25:562–8. 29281157 10.1111/ene.13554

[12] Giovannini G, Bedin R, Ferraro D. et al. Serum neurofilament light as biomarker of seizure-related neuronal injury in status epilepticus. Epilepsia 2022;63:e23–9. 34806176 10.1111/epi.17132 PMC9299158

[13] Velasco R, Villagrán M, Jové M. et al. Encephalitis induced by immune checkpoint inhibitors. JAMA Neurol 2021;78:864. 33720308 10.1001/jamaneurol.2021.0249

[14] Sauer BM, Schmalstieg WF, Howe CL. Axons are injured by antigen-specific CD8+ T cells through a MHC class I- and granzyme B-dependent mechanism. Neurobiol Dis 2013;59:194–205. 23899663 10.1016/j.nbd.2013.07.010 PMC3788647

[15] Nissen MS, Ryding M, Nilsson AC. et al. CSF-Neurofilament light chain levels in NMDAR and LGI1 encephalitis: a National Cohort Study. Front Immunol 2021;12:719432. 10.3389/fimmu.2021.719432. PMC8716734 34975832

[16] Ferreira-Atuesta C, Reyes S, Giovanonni G, Gnanapavan S . The evolution of neurofilament light chain in multiple sclerosis. Front Neurosci 2021;15:642384. 10.3389/fnins.2021.642384. PMC8055958 33889068

